# Rapid culture‐based detection of *Legionella pneumophila* using isothermal microcalorimetry with an improved evaluation method

**DOI:** 10.1111/1751-7915.13563

**Published:** 2020-03-25

**Authors:** Christian Fricke, Juan Xu, Feng‐Lei Jiang, Yi Liu, Hauke Harms, Thomas Maskow

**Affiliations:** ^1^ Department of Environmental Microbiology Helmholtz‐Centre for Environmental Research – UFZ Leipzig Germany; ^2^ Key Laboratory of Analytical Chemistry for Biology and Medicine (Ministry of Education) College of Chemistry and Molecular Sciences Wuhan University Wuhan 430072 China

## Abstract

The detection and quantification of *Legionella pneumophila* (responsible for legionnaire's disease) in water samples can be achieved by various methods. However, the culture‐based ISO 11731:2017, which is based on counts of colony‐forming units per ml (CFU·ml^‐1^) is still the gold standard for quantification of *Legionella* species (spp.). As a powerful alternative, we propose real‐time monitoring of the growth of *L*. *pneumophila* using an isothermal microcalorimeter (IMC). Our results demonstrate that, depending on the initial concentration of *L*. *pneumophila*, detection times of 24–48 h can be reliably achieved. IMC may, therefore, be used as an early warning system for *L. pneumophila* contamination. By replacing only visual detection of growth by a thermal sensor, but otherwise maintaining the standardized protocol of the ISO 11731:2017, the new procedure could easily be incorporated into existing standards. The exact determination of the beginning of metabolic heat is often very difficult because at the beginning of the calorimetric signal the thermal stabilization and the metabolic heat development overlap. Here, we propose a new data evaluation based on the first derivation of the heat flow signal. The improved evaluation method can further reduce detection times and significantly increase the reliability of the IMC approach.

## Graphical abstract

Based on isothermal microcalorimetry, a method is developed that allows the rapid detection and quantification of *Legionella pneumophilia* while maintaining the principle of the culture‐based standard (ISO 11731:2017). Our results demonstrate that, depending on the initial concentration of *L.* *pneumophila*, detection times of 24–48 h can be reliably achieved in contrast to the conventional method, which takes between 72 and 240 h.

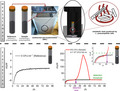

## Introduction

Legionnaire's disease, caused by pathogenic bacteria of *Legionella* spp., manifests itself as an atypical form of pneumonia (Fraser *et al.*, 1977; McDade *et al.*, 1977; Fields *et al.*, 2002; Hilbi *et al.*, 2010). Besides this, a less dangerous, non‐pneumonic form, the so‐called Pontiac fever (Rowbotham, 1980a; Tossa *et al.*, 2006; Ward *et al.*, 2010) can be caused by *Legionella* spp.. The best‐characterized species of the genus is *Legionella pneumophila*, which comprises 16 serogroups (Kazandjian *et al.*, 1997; Yu *et al.*, 2002; Aurell *et al.*, 2003). It is a Gram‐negative bacterium that is omnipresent in the environment, especially in aquatic systems (Rowbotham,1980b). Here, amoebae act as host cells for *L. pneumophila* to protect and multiply them (Rowbotham, 1980b; Rowbotham, 1983; Thomas *et al.*, 2010). However, the greatest danger is posed by *L. pneumophila* when it invades technical water systems (private and public buildings), air conditioners, cooling towers, etc. (Fliermans *et al.*, 1981; Orrison *et al.*, 1981; Stout *et al.*, 1985; Fields *et al.*, 2002). The transmission occurs by inhalation of small water droplets containing *L. pneumophila* (Bollin *et al.*, 1985). The aerosols are then able to enter the respiratory tract and infect human macrophages in which *L. pneumophila* multiplies. Finally, the human macrophages are killed and the replicated *L. pneumophila* bacteria are released and can attack further macrophages (Segal and Shuman, 1999; Khweek and Amer, 2010).

Since the first recognition of *L. pneumophila* in 1976, several methods were developed for the detection of this pathogen (Villari *et al.*, 1998; Díaz‐Flores *et al.*, 2015; Mobed *et al.*, 2019). One of the earliest diagnostic tools was based on immunofluorescence labelling (Cherry *et al.*, 1978; Berdal *et al.*, 1979). The semi‐selective nutrient medium developed by Edelstein and co‐workers in 1979, based on charcoal yeast extract (CYE) agar allowed the first selective enrichment of *L. pneumophila* cultures from contaminated specimens (Edelstein and Finegold, 1979). Later, more sensitive and selective nutrient media were explored (Warren and Miller, 1979; Ristroph *et al.*, 1980), which are admixed with antibiotics (e.g. as Glycine Vancomycin Polymyxin Cycloheximide (GVPC) agar) to suppress the growth of accompanying microorganisms (Edelstein 1981). The common present‐day practice is to determine the number of *L. pneumophila* cells in environmental samples as prescribed in the standardized protocol of the ISO 11731:2017. This ISO method describes standardized cultivation of *L*. *pneumophila* on selective culture media (GVPC Agar). The degree of contamination is determined quantitatively as CFU per 100 ml sample.

Besides this, there are several other techniques known for the detection of *L. pneumophila*: for instance, urinary antigen tests (UAT) (Bibb *et al.*, 1984; Samuel *et al.*, 1988; Dominguez *et al.*, 1996), indirect and direct fluorescent antibody (IDFA) staining (Winn *et al.*, 1980; Makin and Hart, 1989; Alary and Joly, 1992), a high number of different qualitative and quantitative polymerase chain reaction (PCR) approaches (Starnbach *et al.*, 1989; Bej *et al.*, 1991; Catalan *et al.*, 1994; Matsiotabernard *et al.*, 1994; MatsiotaBernard *et al.*, 1997; Ballard *et al.*, 2000; van der Zee *et al.*, 2002; Fiume *et al.*, 2005; Boss *et al.*, 2018) and a variety of biosensors like surface plasmon resonance (SPR) immunosensor, electrochemical as well as genosensors etc. (Oh *et al.*, 2003; Li *et al.*, 2012; Mobed *et al.*, 2019). Especially, qPCR techniques are becoming more and more used in practice. One example is the ISO norm (ISO/TS 12869:2019) which deals with the detection and quantification of *L. pneumophila* (Omiccioli *et al.*, 2015). All these diverse techniques are able to detect *Legionella* spp. much faster (minutes to a few hours) compared with the conventional cultivation approach (3–7 days) (Mobed *et al.*, 2019). However, they have in common that expensive agents and a high level of expertise are required for their execution and data interpretation. Therefore, it is not surprising that the cultivation‐dependent detection of *L. pneumophila* is still the gold standard.

For this reason and in contrast to the general research trend towards molecular biological techniques in the field of *L. pneumophila* detection, we propose a culture‐based method using an isothermal microcalorimeter (IMC) to track the growth of *L. pneumophila* in real‐time. We suggest replacing visual detection by the human eye through a highly sensitive thermoelectric device (*i*.*e*. Peltier element) of an IMC and exploit the metabolic heat evolved for early detection of bacterial growth (Wadsö and Goldberg, 2001; von Stockar 2010; Maskow *et al.*, 2014; Braissant et al., 2015a,2015b). Additionally, if IMC experiments are performed on solid medium further inspections like checking the colony morphology after the experiment are possible if glass ampoules are used. Depending on the bacterial strain as well as on the medium this subsequential visual inspection might provide some information on potential accompanying bacterial flora. Like colony counts, IMC recognizes only active and cultivable bacteria as the harmful fraction of the pathogen. Advantageously, IMC is very easy to implement and data interpretation does not require specific expertise. Furthermore, using IMC is fully compatible with ISO 11731:2017 sample preparation. Hence, it can be easily integrated into the existing standard protocols with the added benefit of potentially reduced detection times, especially for high concentrations of *L. pneumophila* (> 10^4^ CFU L^‐1^) (WHO 2007). The three objectives of our study are therefore firstly to show the applicability of calorimetric monitoring to *L. pneumophila* contamination, secondly to optimize the data evaluation and thirdly, to analyse the dependence of the detection time on pathogen concentration.

## Results and Discussion

### Principles of heat flow measurements of bacterial growth

**Fig. 1:**
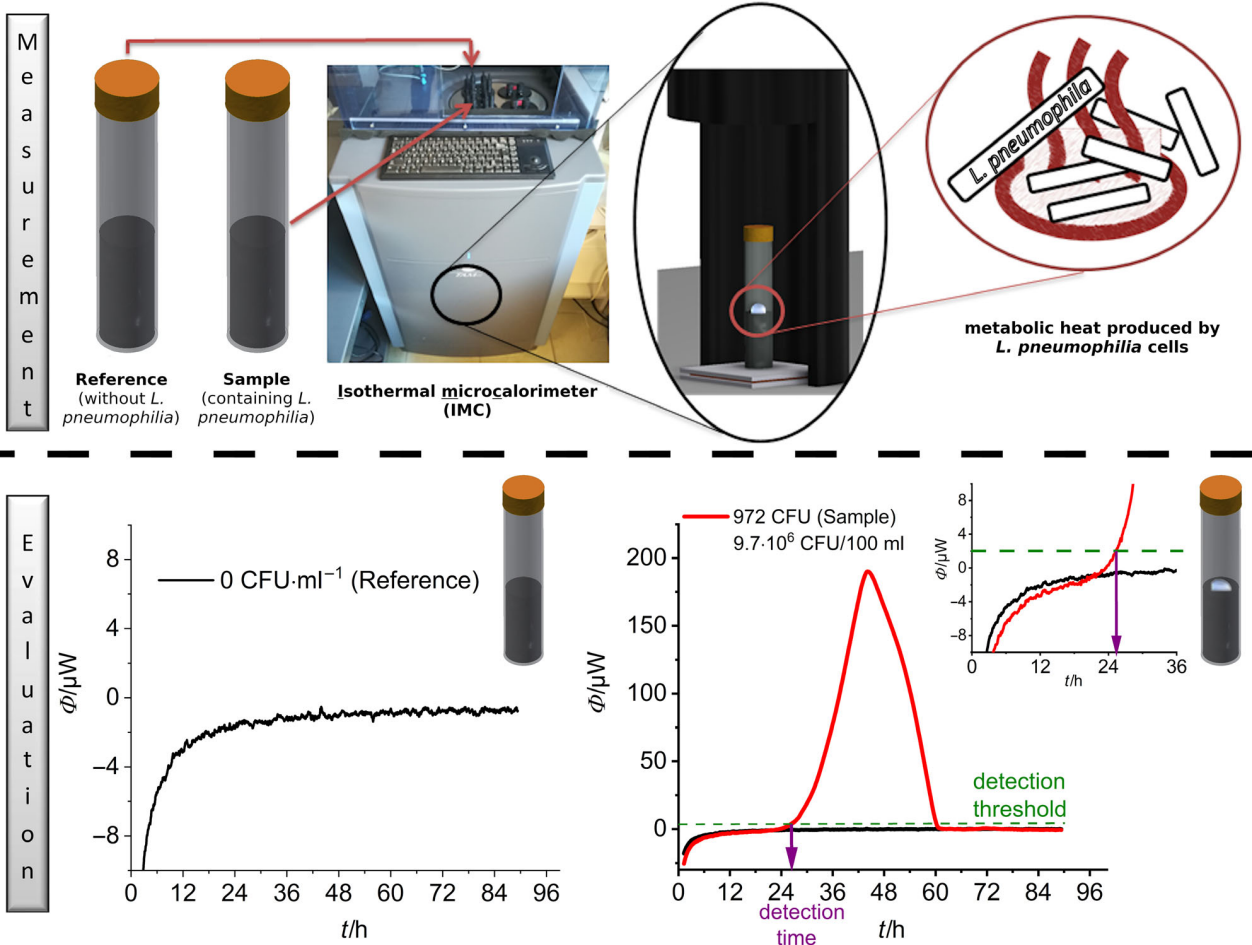
Experimental setup and data evaluation for the microcalorimetric investigation of the growth of *L. pneumophila.*

Medium and sample are held by a sealable glass ampoule (calorimetric vessel) which is put into a calorimeter. The metabolic heat flows via a Peltier element to a thermally controlled heat sink. The Peltier element provides a voltage which is proportional to the metabolic heat production rate *Φ*(*t*) (in W) (see Fig. 1).

The conversion factor of measured voltage to heat output signal is determined by electrical calibration. The actual heat flow *Φ*(*t*) depends on the number of active *L. pneumophila* cells at any point in time *N*(*t*) and the cell‐specific heat evolved by each cell *φ*_0_ (in W) (Eq. 1, Chang‐Li *et al.*, 1988):

(1) $$\Phi \left(t\right)\;=\;N\left(t\right)\cdot \varphi_{0}.$$

It is well known that bacterial growth if all nutrients are available in excess follows the following exponential function:

(2) $$N\left(t\right)\;=\;N_{0}\cdot \exp\left(\mu \cdot t\right).$$

where *N*_0_ is the initial bacterial concentration in the sample under investigation, *µ* is the specific growth rate (in h^‐1^) and *t* the elapsed time (in h). Combining Eqs 1 and 2 leads to:

(3) $$\Phi \left(t\right)\;=\;N_{0}\cdot \varphi_{0}\cdot \exp\left(\mu \;\cdot \;t\right)\;\;\text{or}\;\ln\left(N_{0}\right)\;=\;-\mu \cdot t\;+\;\ln\left(\frac{\Phi \left(t\right)}{\varphi_{0}}\right).$$

The logarithmized form of Eq. 3 makes it possible to establish a linear relationship between the initial concentration *N*_0_ and the time *t* (in h) that passes until a given heat production rate *Φ(t)* is detected. Assuming a certain value as thermal detection limit (e.g. 2 µW) (Braissant *et al.*, 2010), the corresponding time could be called detection time *t*_dect_. It could be shortened by using a more sensitive calorimeter with a smaller thermal detection limit or a higher starting concentration of *L. pneumophila*. However, it has to be noted that not all commercially available microcalorimeters achieve this thermal detection limit. Nevertheless, the latter can be achieved by concentrating the sample, for example by membrane filtration. Membrane filtration of 100 ml sample volume is also used for the ISO 11731:2017 standard. When interpreting the results of plating 10 µl (in the case of the IMC experiments), the enrichment by a factor of 10^4^ should be kept in mind. The *t*_dect_ is used to calculate the number of *L. pneumophila* cells in the calorimetric vessel and by considering the enrichment in the sample according to Eq. 3.

**Fig. 2:**
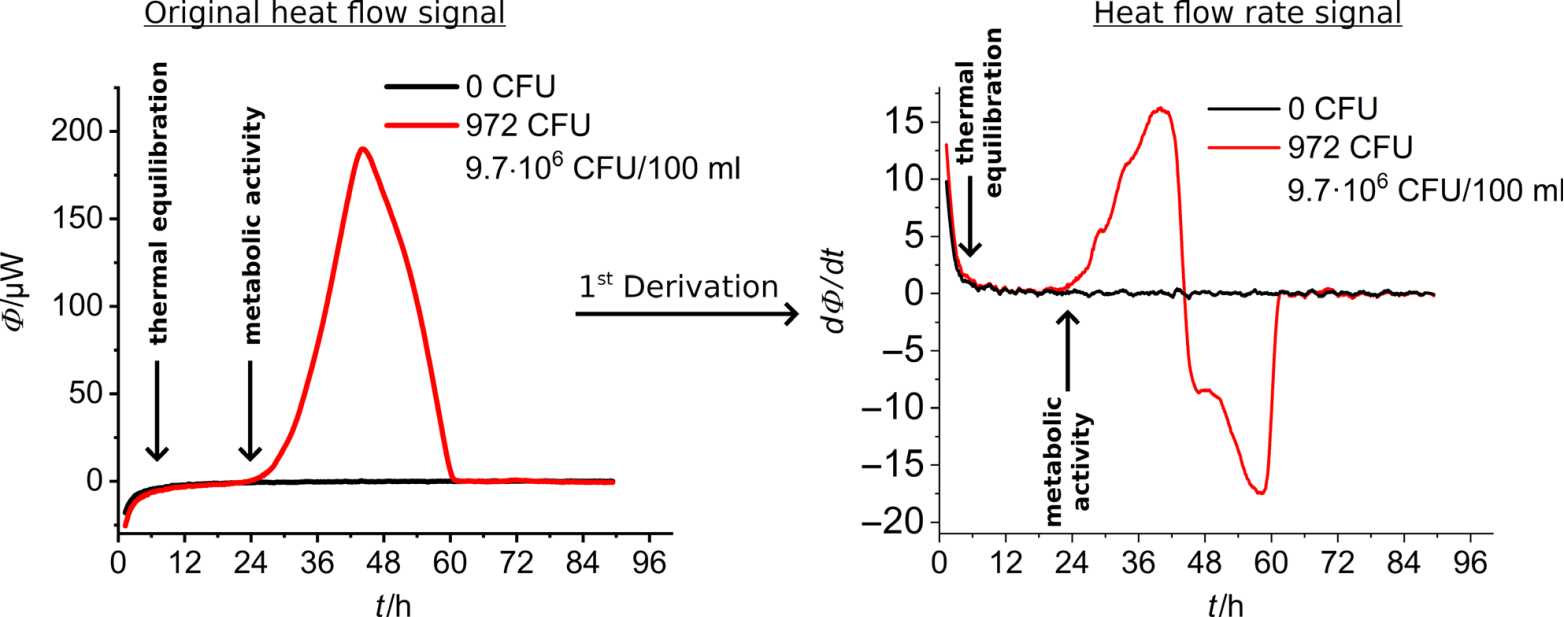
Derivation of the original heat flow signal. Left: First derivation of the heat flow after the time (heat flow rate, ϕ). Right: The original signal, the red line stands for a metabolically influenced heat signal and the black line for the blank.

The thermal signal is essentially determined by two counteracting effects (see also Supporting information). First, the ampoule is entering the calorimeter with a temperature *T*_0_ slightly deviating from the calorimeter temperature *T*_C_ and needs to be thermally equilibrated (typically an endothermal signal). Second, heat is evolved due to metabolic activity (exothermal signal). As a result of these two effects, there is a shift in the baseline, which is clearly noticeable at the beginning of all measurements. To ensure a reliable determination of the detection time, additional baseline corrections are necessary. Another possibility, which to our knowledge has never been tested before in measuring metabolic activity, is the analysis of the first derivative of the heat flow (see Fig. 2), which potentially eliminates the shift of the baseline and allows to distinguish the physical from the metabolic effect. In other words, such an approach does not require arbitrary baseline correction.

**Fig. 3:**
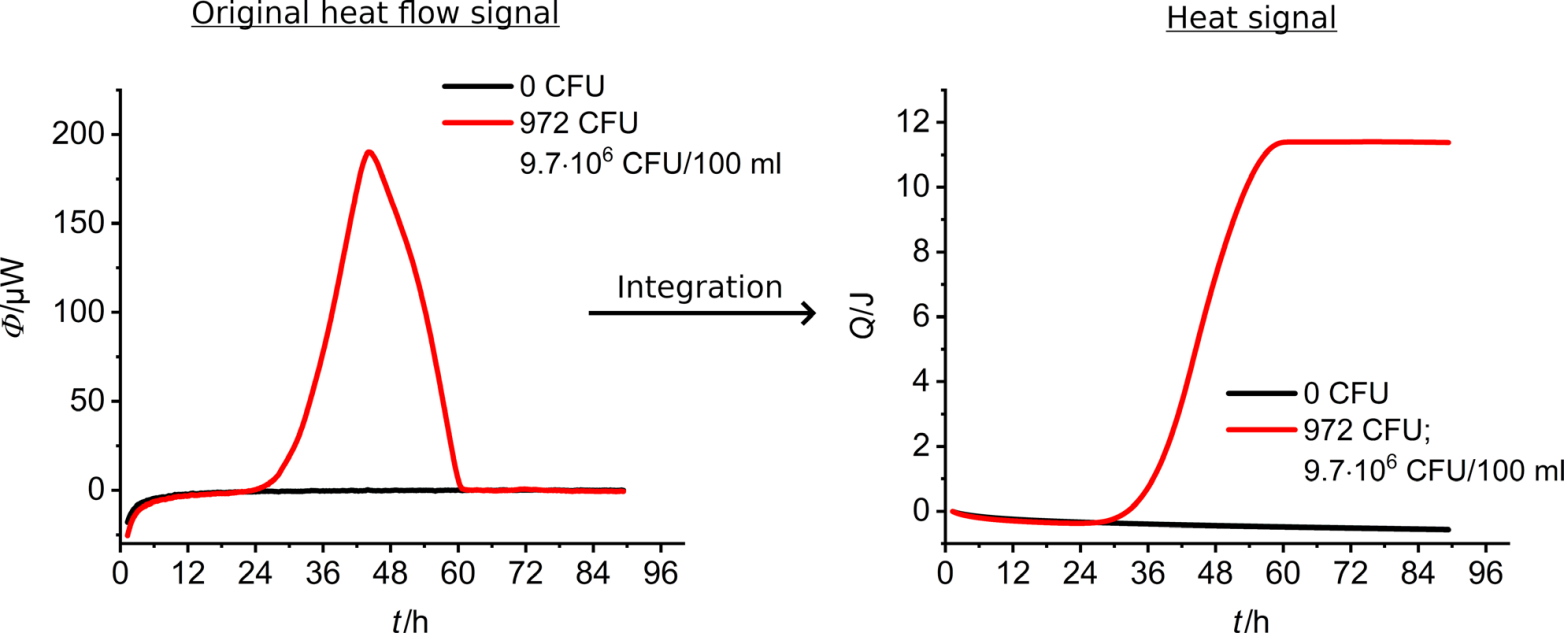
Integration of the original heat flow signal. Left: Integral of the heat flow (total heat, *Q*). Right: The original signal, the red line stands for a metabolically influenced heat signal and the black line for the blank.

If necessary, additional information about the growth kinetic of *L*. *pneumophila* can be obtained via integration of the heat flow signal. The integrated heat flow shown in Fig. 3 follows a classical sigmoidal bacterial growth curve, which can be divided into lag phase, exponential growth phase and stationary phase (Monod, 1949).

However, more sophisticated models like the Gompertz equation (Eq. 4) describe lag phase and stationary phase and allows to derive important kinetic growth parameter from the observed integrated heat flow curve (Braissant *et al.*, 2013).

(4) $$Q\left(t\right)\;=\;Q_{\max}\cdot \exp\left(-\exp\left(-\mu_{\max}\cdot \left(t-\gamma \right)\right)\right).$$

In this equation, *Q*_max_ (in J) represents the total amount of heat evolved during the growth. The parameter *µ*_max_ (in h^‐1^) corresponds to the maximum growth rate during the exponential phase and *γ* (in h) corresponds to the duration of the lag phase, that is the lag time.

### Applicability of L.  pneumophila detection by IMC measurement

**Fig. 4:**
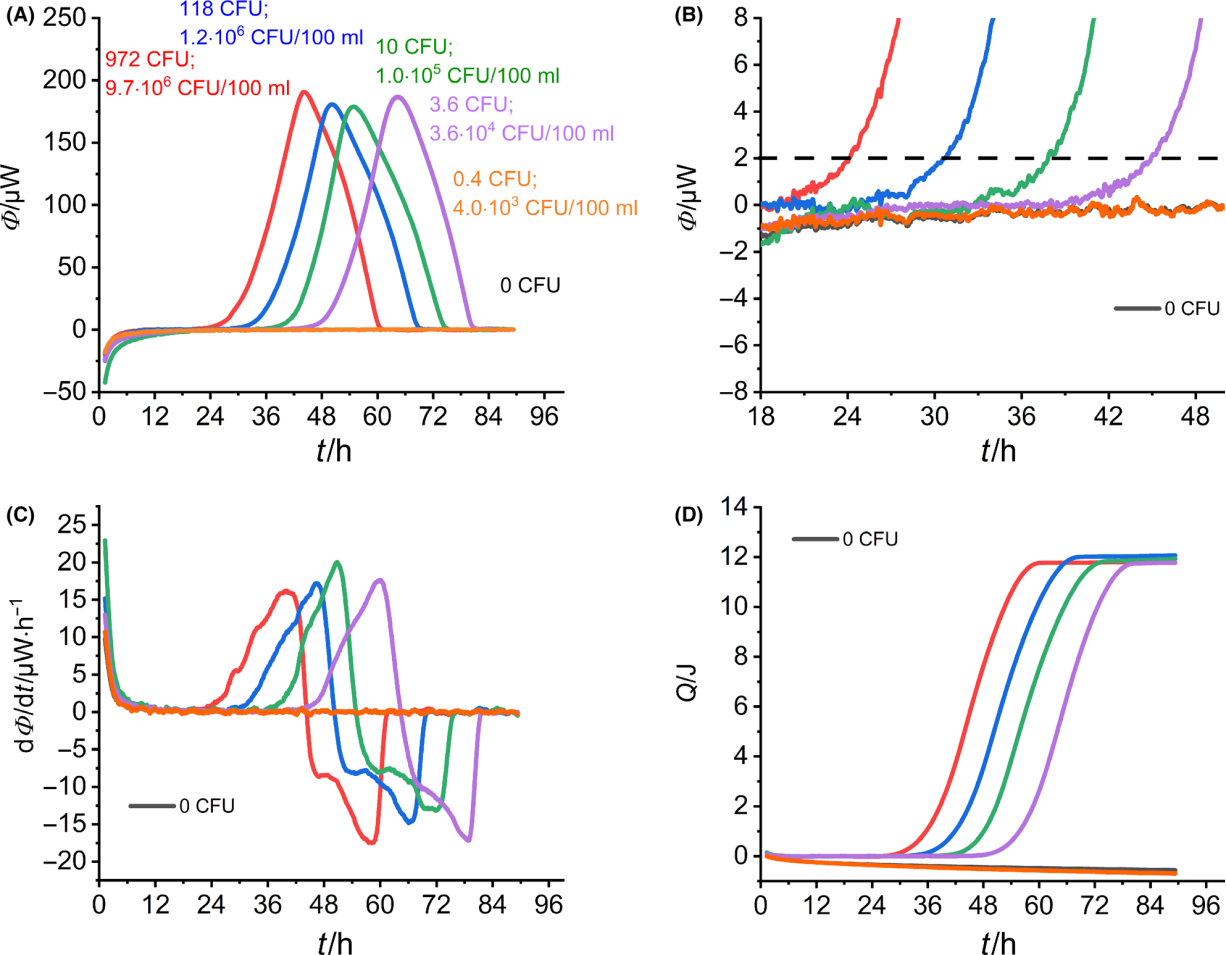
Summary of the IMC monitoring of the growth of *L. pneumophila*. A. Heat flow over time depending on the initial bacterial concentration. B. Magnification of the heat flow near the threshold value. C. First derivatives of the heat flow signals depending on the initial bacterial concentration. D. Integrals of the heat flow signals depending on the initial bacterial concentration.

At first, the calorimetric signal has to be interpreted (Fig. 4). All initial bacterial concentrations are given in CFU (number of bacteria per ampoule) and CFU/100 ml (as specified by ISO). Independent of the initial number of *L. pneumophila* cells, the heat flow signals had the same shape and displayed only a single peak. After reaching the maximum, all heat flow signals returned to the baseline (Fig. 4A). With increased initial bacterial concentration, the heat flow signals appeared earlier, according to Eq. 3.

Braissant *et al.* (2010) obtained similar results with their concentration‐dependent investigation of *Mycobacterium tuberculosis*. The lowest initial concentration (0.4 CFU per calorimetric ampoule) did not result in an increase in the heat flow signal. This can be explained by the inoculum volume of 10 µl, which statistically contains only approx. 0.1 CFU so that only one CFU in every tenth sample is expected. To determine a corresponding detection time for each heat flow signal, we set the threshold value at *Φ*(*t*) = 2 µW according to literature recommendations (Braissant *et al.*, 2010). If the respective heat flow signal reaches this value, the detection time can be read off directly (Fig. 4B).

If one considers the beginning of the heat flow signals, a sharp increase in all signals was observed (Fig. 4A). All heat flows showed the transient process from thermal equilibration of the calorimetric vessel to a metabolic heat signal. This kind of transitions led apparently to strong baseline drifts at the beginning of the signal and complicated the whole data evaluation because arbitrary baseline corrections are necessary to quantify the corresponding detection times correctly. In order to avoid baseline corrections, we calculated the first derivative of the heat flow signals (Fig. 4C). The first derivative provided a good baseline (the flat line linking the two effects).

Further information (such as maximum specific growth rate, total heat generated and the duration of the lag phase) can be derived from the integration of the heat flow signal and the parameter adjustment to the Gompertz model (eq. 4, see Supporting information). The integrated heat curves can be seen in Fig. 4D. The integrated curves show typical sigmoidal behaviour. The exponential part corresponds to the growth/proliferation phase of the bacteria. Reaching the plateau indicates that the existing media (e.g. oxygen and substrates) are depleted.

### Improved data evaluation

**Fig. 5:**
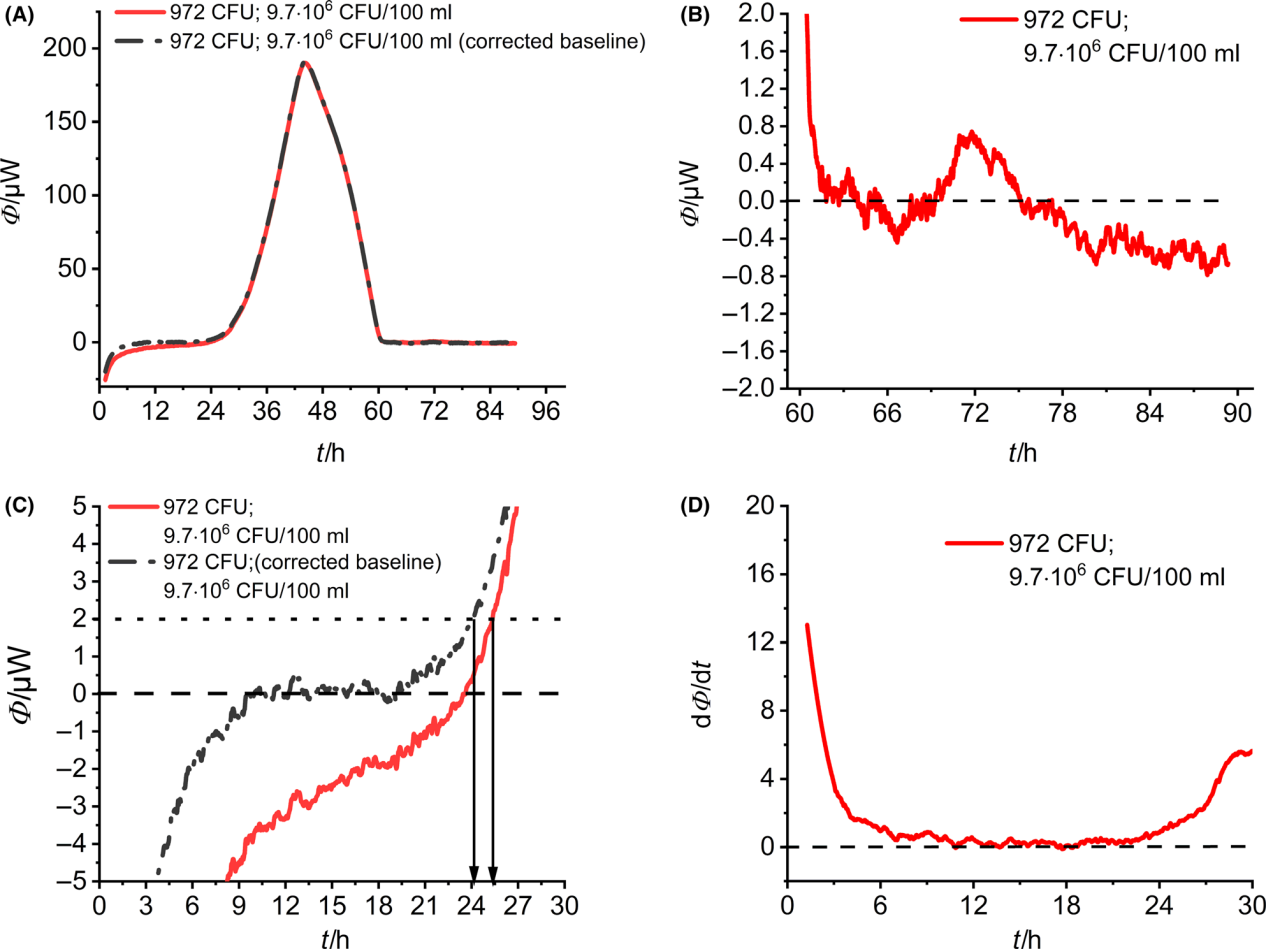
Problems and improvements in data evaluation. A. Total heat flow over time without (red) and with baseline corrections (black). B. End of the heat flow signal. C. Beginning of the heat flow signal. Due to incomplete thermal equilibration, the beginning suggests negative heat flow. The black curve with baseline correction results in a detection time of 24.1 h, whereas the uncorrected red curve gives a longer detection time of 25.3 h. D. The first derivation of the heat flow signal obviating the need for baseline correction.

To illustrate the effect of baseline correction, Fig. 5A shows the uncorrected (red) and baseline‐corrected (black) heat flow signal. The interfering endothermal signal obviously has a significant influence on the definition of the baseline and thus on the detection time.

The uncorrected baseline reaches the threshold value (2 µW, dotted line) approx. 1.5 h later than the baseline‐corrected signal. If a linear baseline is to be assumed, two points are needed where the heat production rate is set to zero. One point is obviously after the metabolism is finished. Here, a final signal appeared, which is approximately in the expected range of 0 µW with a signal noise of approx. (± 0.2 µW) as described for the used calorimeter type (TAM III, http://www.tainstruments.com) (Fig. 5B). The other point should be the beginning of the metabolically determined heat flow signal. However, it is difficult to define this point because the signal is interfered by the thermal equilibration (Fig. 5C). The data evaluation proposed by us, simple to practice, considers the first derivative of the total signal with both effects (thermal equilibration and metabolic activity) (Fig. 5D). The result is a broad minimum between the two effects, which can be taken to define a comparatively constant baseline. Due to the on‐line character of the heat signal, the measuring points can be placed very tightly, which enables good signal smoothing (Savitzky–Golay filter, 200 measuring points) and thus good differentiation.

### Dependence of the detection speed on L. pneumophila concentrations

**Fig. 6:**
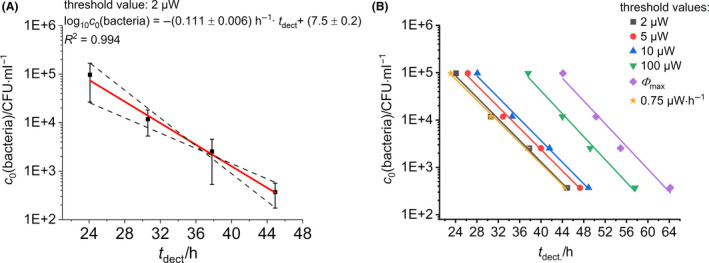
Summary of the data evaluation of the concentration‐dependent measurement. A. Best‐fit line with border‐straights (lower and upper limit of the slope) plotted by the initial bacterial concentration and the detection time for a threshold value at 2 µW. B. Best‐fit lines for different threshold values.

We obtained a linear relationship when plotting the detection time against the logarithm of the initial bacterial concentration (Fig. 6A) according to Eq. 3.

Using this correlation, it is now possible to determine the initial bacterial concentration for each unknown sample of *L. pneumophila*. In order to demonstrate that this linear relationship is not a question of the threshold definition, we additionally determined the corresponding detection times at different threshold values *Φ*(*t*) = 5, 10, 100 µW as well as at the maximum of the heat flow signal *Φ*_max_. The results are summarized in Fig. 6B and all thresholds show the aforementioned linear dependency. To demonstrate that the improved data evaluation might ease the whole evaluation process, the detection time was also determined for the first derivative of the respective heat flow signals (threshold: 0.75 µW h^‐1^). In this way, a reduced detection time is obtained (approx. 1 hour).

### Characteristic quantities derived from heat flow measurements

All information received from Fig. 4 is compiled in Table 1. A comparison of the specific growth rates shows that there is no significant relationship between the growth rate and the initial bacterial concentration according to the expectations expressed in Eq. 3. Based on the growth rates, the doubling times *t*_d_ between 4.2 and 4.6 h were calculated. These doubling times are in good agreement with literature data (4 to 6 h) (Donohue *et al.*, 2014). The total amount of heat can easily be derived from the integral of the heat flow signals (Fig. 4D). The magnitude of *Q*_max_ can be estimated by using the oxycaloric equivalent $\Delta_{k}\cdot H_{O_{2}}$ = – (455 ± 25) kJ·mol^‐1^ O_2_ (Gnaiger and Kemp, 1990). The volume of oxygen in the headspace was approx. 3 ml. The theoretical heat evolved during aerobic growth is (11.2 ± 0.6) J and thus in good accordance with our finding of 12.0 to 12.4 J (for the calculation, see Supporting information). The lag time *γ* correlates indirectly with the initial bacterial concentration. The parameter of the Gompertz equation might be a powerful tool for the quantification of the susceptibility of *L. pneumophila* to antibiotics or biocides. Energy‐dependent efflux pumps, for example should express themselves in a change of *Q*_max_, while bacteriostatic agents should be recognizable by an extension of the lag time *γ* and a reduction of the specific growth rate *µ*_max_.

**Table 1:** Summary of the derived data from the heat flow measurements.

	972 CFU^b^; 9.7 × 10^6^ CFU/100 ml^c^	118 CFU; 1.2 × 10^6^ CFU/100 ml	10 CFU; 1.0 × 10^5^ CFU/100 ml	3.6 CFU; 3.6 × 10^4^ CFU/100 ml
Integral				
*µ*_max_/h^‐1^	0.1650 ± 0.0005	0.1496 ± 0.0004	0.1500 ± 0.0002	0.1507 ± 0.0004
*Q*_max_/J	11.97 ± 0.01	12.33 ± 0.01	12.23 ± 0.01	12.41 ± 0.01
γ/h	42.65 ± 0.01	49.25 ± 0.01	54.87 ± 0.01	62.93 ± 0.01
Measured data				
*Φ*_max_(*t*)/µW	190.7 (44.1 h)	180.3 (50.3 h)	178.9 (54.9 h)	186.6 (64.2 h)
*t*_dect_ ^a^	24.1	30.6	37.8	44.9

a Threshold value: 2 µW.

b Number of bacteria per ampoule.

c Enrichment due to the ISO 11731:2017 specification.

The maximum heat flow demonstrates that only small amounts of medium (1000 µL) and a small volume of air (3000 µl) are sufficient for these fastidious bacteria to grow in such a way that enough heat is evolved, to be detected even by less sensitive calorimeters. The greatest benefit of the calorimetric method is faster detection (24 to 45 hours depending on initial bacterial concentration) than by conventional cultivation on plates (3 to 7 days). Depending on the initial bacterial concentration, detection within one day is also possible. If one considers less sensitive calorimeters, with a thermal detection limit of only 10–100 µW, detection times for 100 CFU/ml between 56 and 63 h will be achieved. IMC experiments do not require counting and handling plates thus led to a reduction in workload. Additionally, closed ampoules support safer handling of pathogenic bacteria (Braissant *et al.*, 2010).

## Conclusion

In summary, our data demonstrate that IMC might be a powerful analytic tool for fast and reliable detection of *L. pneumophila*. Critical concentrations (100 CFU/ ml) can be detected after two days and concentrated samples (> 10^6^ CFU/100 ml) resembling harmful doses can be detected within a few hours up to one day. Regarding the analytical procedures of *L. pneumophila*, an on‐line warning might be easily integrated when using microcalorimeters for detection. In addition to the short detection times, the main advantages of calorimetric detection are easy handling and easy data evaluation. From a legal point of view, it might be important for approval that our method is consistent and compatible with ISO 11731:2017 protocol as only the detection deviates.

Isothermal microcalorimetry, a non‐specific method, becomes highly selective through the application of chemical and thermal pre‐treatments (Leoni and Legnani, 2001) and the use of selective media (Descours *et al.*, 2014). The application of membrane filtration as an enrichment process and subsequential cultivation with the aim of shortening detection times was firstly demonstrated in liquid medium by Brueckner and co‐workers using IMC (Brueckner *et al.*, 2017). Recently, heat flow measurements demonstrating the potential of membrane filtration were performed on solid medium after enrichment via membrane filtration for anaerobic cultures (Fricke *et al.*, 2019). In the case of *L. pneumophilia* additionally, the availability and the transport of oxygen from the gas to the liquid phase have to be considered because the calorimetric vessel is usually airtight closed and unstirred. However, from first experiments with *P. putida* mt‐2 KT2440 we know that the membrane technique also works for aerobic cultures in liquid and on solid media (unpublished results). In the face of *Legionella* detection, this might be of significance since the ISO 11731:2017 prescribe for samples containing low initial number of *Legionella* enrichment by membrane filtration.

Uncertainties in the definition of the thermal threshold can be overcome by evaluating the 1st derivative of the heat flow might instead of the thermal signal itself.

In order to make this technology applicable for microbiological‐analytical purposes, three main practical aspects are important: First, microcalorimeters have to be developed specifically for clinical microbiological issues. For instance, conventional IMC cover a large temperature range (e.g. 15–150°C in case of the TAM III, 5–90°C in case of TAM Air (http://www.tainstruments.com), 5–70°C in case of BioCal 2000/4000 (http://www.calmetrix.com)) at the expense of a high price of the instrument or limited thermal sensitivity. For most clinical applications, a narrow temperature range around 37°C will be sufficient improving these specifications. This idea has already been taken up, during the development of a multichannel instrument like the calScreener^TM^ (37°C, http://www.kan-tht.com/images/pdf/calScreener4page.pdf). Second, the small tubes usually applied in calorimetry differ in size and sample vessel surface considerably from conventional Petri dishes, which allow an easy follow‐up analysis of the colony material. Third, the throughput of conventional calorimeters still does not meet the requirements of commercial laboratories (Maskow *et al.*, 2012; Braissant et al., 2015a,2015b). A calorimeter with a moderate price that allows the application of conventional cultivation containers and high‐throughput measurements would certainly find broad acceptance for *L. pneumophila* analysis, particularly since calorimetry complies with the enrichment and cultivation conditions fixed in the ISO 11731:2017.

## Experimental procedure

*Legionella pneumophila* ATCC 33152 (Guangdong Microbial Culture Center, GDMCC, Guangdong, China) was used for the calorimetric investigation. The strain was cultivated on BCYE (buffered charcoal yeast extract) medium, which is composed of (in g l^‐1^): charcoal (2), yeast extract (10), agar (15), ACES buffer (5), KOH (1.4), iron pyrophosphate (0.125), potassium‐α‐ketoglutarate (2) and l‐cysteine hydrochloride (0.2). The pH value was stabilized to 6.8 ± 0.5 by the ACES buffer. A few colonies of a pre‐grown Petri dish were used for liquid pre‐culture of *L*. *pneumophila* on BYE‐broth (without charcoal), which was incubated overnight at 37.0 ± 0.2 °C (HQ45Z, Zhongke Scientific Instrument and Technology Development Co. Ltd., Wuhan, China) and cells were harvested immediately before the calorimetric experiment was performed. The identity of the species was regularly checked by the morphology of the colonies.

The calorimetric measurements were performed in 4 ml glass ampoules in a high‐performance IMC (TAM III, TA Instruments, New Castle, DE, USA). The ampoules and caps were autoclaved at 121°C for 40 min and then filled with 1000 µl warm (~ 50°C), molten BCYE‐agar medium, closed and stored at 4°C. The prepared glass ampoules with the solid media were cooled down to room temperature before the bacteria were added to the medium. Meanwhile, starting from the pre‐culture (OD_600_ = 0.18, 1:10 dilution) a dilution series was performed in 1:10 dilution steps (dilution factor *f*_a_ = 10^5^–10^8^). 10 µl of each dilution step was added into a glass ampoule. After adding the bacteria, the glass ampoules were closed and set into a pre‐heating position (to reach thermal equilibrium) for 15 min in the IMC. In the last step, the glass ampoules were pushed into the measuring position and after further 45 min (to reach thermal equilibrium), the heat flow signal was recorded.

## Conflict of interest

The authors declare that the research was conducted in the absence of any commercial or financial relationships that could be construed as a potential conflict of interest.

## Supporting information


**Fig. S1**. Heat over time diagram. The black line describes the integrated heat signal determined by metabolic activity. The red line shows the Gompertz fit.
**Fig. S2**. The schematic structure of the ampoules for monitoring *L. pneumophila *growth.
**Table S1**. Summary of the physical quantities for calculating the total heat production.
