# Supplementary material for: Rapid culture‐based detection of Legionella pneumophila using isothermal microcalorimetry with an improved evaluation method
Source: Microb Biotechnol. 2020 Mar 25;13(4):1262–72. doi: 10.1111/1751-7915.13563 (PMC7264898; doi:10.1111/1751-7915.13563)
Supplement: Supplementary file 1 — Fig. S1. Heat over time diagram. The black line describes the integrated heat signal determined by metabolic activity. The red line shows the Gompertz fit. Fig. S2. The schematic structure of the ampoules for monitoring L. pneumophila growth. Table S1. Summary of the physical quantities for calculating the total heat production. [file MBT2-13-1262-s001.docx]

**Rapid culture-based detection of *Legionella* *pneumophila* using isothermal microcalorimetry with an improved evaluation method**

Christian Fricke^1^, Juan Xu^2^, Feng-Lei Jiang^2^, Yi Liu^2^, Hauke Harms^1^, Thomas Maskow^1*^

^1^Helmholtz-Centre for Environmental Research – UFZ, Department of Environmental Microbiology, Leipzig, Germany

^2^Key Laboratory of Analytical Chemistry for Biology and Medicine (Ministry of Education), College of Chemistry and Molecular Sciences, Wuhan University, Wuhan, 430072, China.

For correspondence:

*E-Mail: Thomas.Maskow@ufz.de, Tel. +49341 2351328

Contents

[1. Thermal behavior of the calorimetric ampoule 1](#_Toc22547735)

[2. Gompertz-Fit 2](#_Toc22547736)

[3. Estimation of the total heat using the oxycaloric equivalent 3](#_Toc22547737)

[Reference 4](#_Toc22547738)

# Thermal behavior of the calorimetric ampoule

At the begin of the growth process, the metabolic heat evolution *P*_M_ can be described by **eq. (1)**.

$P_{M}=P_{0}\cdot e^{\mu\cdot t}$ (1)

Here is *P*_0_ the metabolic start activity (in W) which is proportional to the number of active cells and *µ* is the specific growth rate (in s^-1^). The resulting heat is dissipated via a Peltier element with the area *A* (in m^2^) and the heat conduction coefficient *λ* (in W m^-1^ K^-1^) and measured as a voltage proportional to (T-T_C_).

$P=A\cdot\lambda\cdot\left( T-T_{C} \right)$ (2)

*T, T_C_* stand for temperature in K and temperature of the calorimeter, respectively. The entire process is described by a heat balance equation (the calorimeter equation) **(3)**.

$V_{r}\cdot\rho\cdot c_{p}\cdot\frac{d T}{d t}=P_{0}\cdot e^{\mu\cdot t}-A\cdot\lambda\cdot\left( T-T_{C} \right)$ (3)

*V_r_, ρ, c_P_, t* stand for the filling volume of the ampoule (in m^3^), the density of the medium (in kg m^-3^), the specific heat capacity (in J kg^-1^ K^-1^) and the time (ins), respectively.

# Gompertz-Fit

By integrating the received heat flow signals, the total heat of the growth can be obtained. The resulting heat curve showed classical sigmoidal behavior. Using logistic equations like the Gompertz function, parameters such as *Q*_max_, the saturation limit, *µ*_max_, the maximum slope of the curve and *γ*, refers to the shift along the time-axis can be obtained. The Gompertz fit and the heat integral are shown in **Fig. S1**.


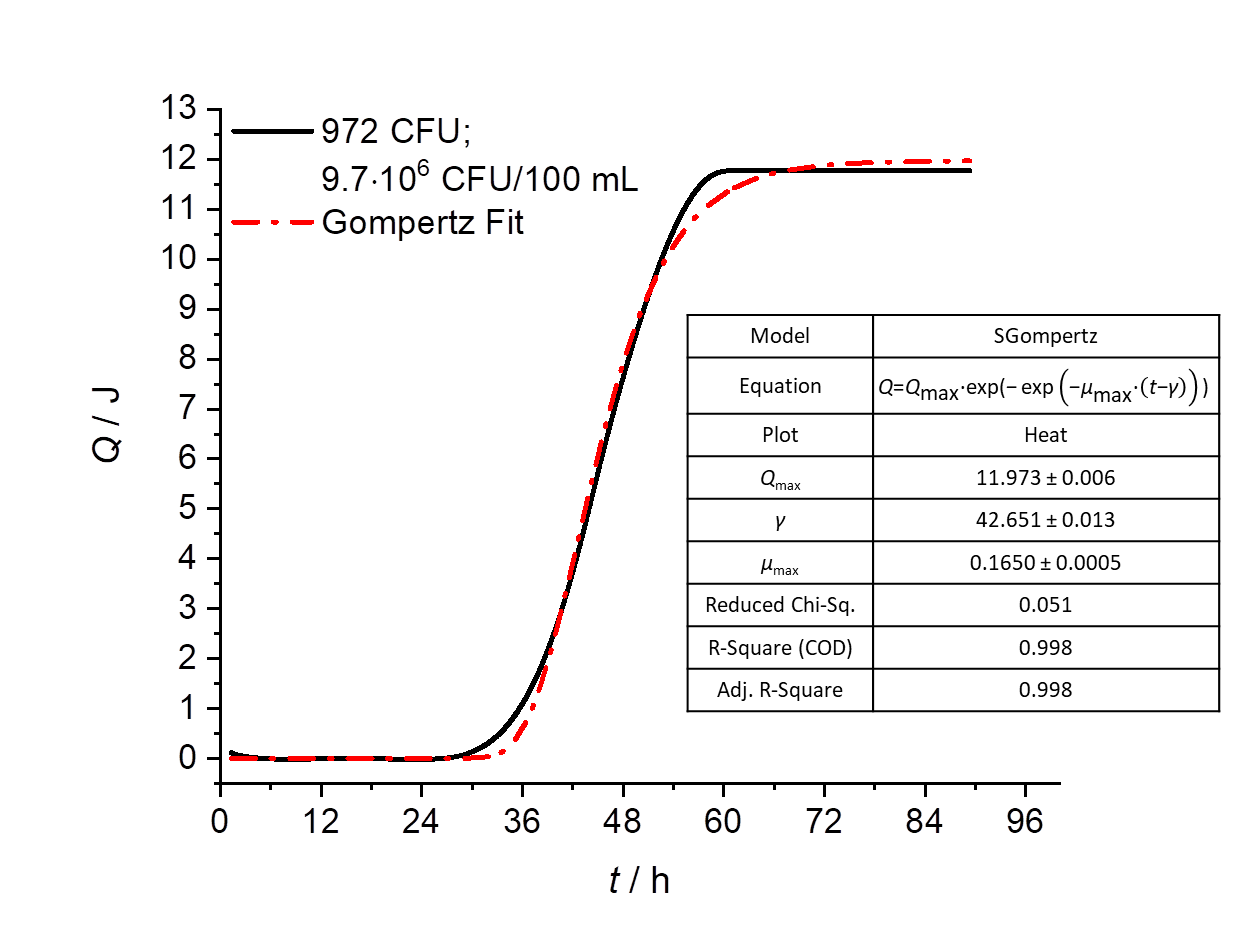


**Figure S1:** Heat over time diagram. The black line describes the integrated heat signal determined by metabolic activity. The red line shows the Gompertz fit.

The fit showed a high correlation (*R*^2^ = 0.998) with the integrated heat signal. Microbiological quantities can be assigned to the individual parameters from the Gompertz Fit. The parameters obtained from the fit are *Q*_max_ = (11.97 ± 0.01) J, *γ* = (42.65 ± 0.01) h and *µ*_max_ = (0.1650 ± 0.0005) h^-1^. *Q*_max_ represents the total heat. *µ*_max_ the maximum growth rate of the respective bacteria and *γ* the lag time of bacterial growth.

# Estimation of the total heat using the oxycaloric equivalent

The initial situation is illustrated in **Fig. S2**. The sample contained 1 mL substrate and 3 mL air in headspace.


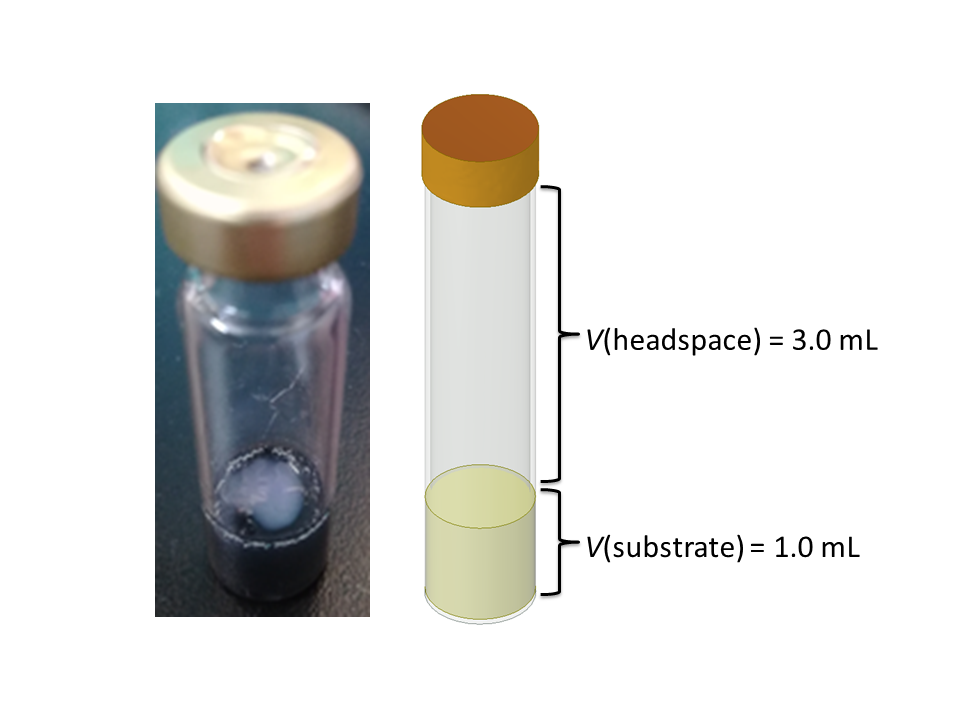


**Figure S2:** The schematic structure of the ampoules for monitoring *L. pneumophila* growth.

The following data are necessary for calculating the total heat production of *L. pneumophila* during aerobic growth.

Table 1: Summary of the physical quantities for calculating the total heat production

| parameter | value | dimension | Reference |
| --- | --- | --- | --- |
| *V*(air) | 0.003 | L | - |
| *ρ*(air) | 1.1381^a^ | kg·m³ | - |
| *w*(O_2_) | 23.14 | % | (Saha, 2008) |
| *M*(O_2_) | 32 | g·mol^-1^ | - |
| Δ_k_$\text{H}_{\text{O}_{\text{2}}}$ | (455 ± 25) | kJ·mol^-1^ O_2_ | (Gnaiger and Kemp, 1990) |

^a^ determined by the specific gas constant of dry air, *R*_s_ = 287.058 J·kg^-1^·K^-1^ at 310.15 K

The mass of air and molecular oxygen can be calculated as follow:

$m\left( \mathrm{air} \right)=V\left( \mathrm{air} \right)\cdot\rho(\mathrm{air})$ (4)

$m\left( O_{2} \right)=w\left( O_{2} \right)\cdot m(O_{2})$ (5)

The molar mass of molecular oxygen can be used to calculate the amount of molecular oxygen in the headspace.

$n\left( O_{2} \right)=\frac{m(O_{2})}{M\left( O_{2} \right)}$ (6)

In the last step, the total heat released from the aerobic growth can be calculated using the oxycaloric equivalent Δ_k_$\text{H}_{\text{O}_{\text{2}}}$.

$Q=n\left( O_{2} \right)\cdot\Delta_{k}H_{O_{2}}$ (7)

The theoretical heat evolved during aerobic growth under the circumstances is *Q* = (11.2 ± 0.6) J.

# Reference

Gnaiger, E., and Kemp, R.B. (1990). Anaerobic metabolism in aerobic mammalian cells: information from the ratio of calorimetric heat flux and respirometric oxygen flux. *Biochimica et Biophysica Acta (BBA) - Bioenergetics* 1016**,** 328-332.

Saha, K. (2008). *The earth's atmosphere - its physics and dynamics.* Berlin: Springer-Verlag Berlin Heidelberg.
